# Mental Health Consequences of the COVID-19 Outbreak Among Emergency Department Healthcare Workers

**DOI:** 10.1155/2024/8871959

**Published:** 2024-09-09

**Authors:** Marion Douplat, Fabien Subtil, Anne Termoz, Laurent Jacquin, Frédéric Verbois, Veronique Potinet, Romain Hernu, Verena Landel, Stéphanie Mazza, Julien Berthiller, Julie Haesebaert, Karim Tazarourte

**Affiliations:** ^1^ Emergency Department Hospices Civils of Lyon Lyon Sud Hospital, Pierre Bénite F-69495, France; ^2^ Research on Healthcare Performance (RESHAPE) Université Claude Bernard Lyon 1, Lyon, France; ^3^ UMR ADéS 7268 Aix-Marseille University/EFS/CNRS Espace éthique méditerranéen, Marseille, France; ^4^ Service de Biostatistique Hospices Civils de Lyon, Lyon, France; ^5^ Université de Lyon Université Lyon 1 CNRS Laboratoire de Biométrie et Biologie Évolutive, UMR 5558, Villeurbanne, France; ^6^ Pôle de Santé Publique Service de recherche et d'épidémiologie cliniques Hospices Civils de Lyon, Lyon, France; ^7^ Emergency Department Hospices Civils of Lyon Edouard Herriot Hospital, Lyon F-69003, France; ^8^ Emergency Department Villefranche Hospital, Gleize F-69400, France; ^9^ Emergency Department Hospices Civils of Lyon Croix Rousse Hospital, Lyon F-69004, France; ^10^ Direction de la Recherche en Santé Hospices Civils de Lyon, Lyon, France; ^11^ CNRS, INSERM Centre de Recherche en Neurosciences de Lyon CRNL U1028 UMR5292, F-69500, Bron, Lyon, France

**Keywords:** burnout, fatigue, paramedics, physicians, sleepiness

## Abstract

**Study Objective:** The present study is aimed at providing an assessment of the changes in burnout, job strain, isostrain, sleepiness, and fatigue levels over time and identifying factors associated with these symptoms among healthcare workers in French emergency departments (EDs).

**Method:** We conducted a prospective, multicenter study in four EDs and an emergency medical service. Participants completed questionnaires at inclusion and at 90 days to assess burnout, job strain, isostrain, sleepiness, and fatigue.

**Results**: A total of 211 respondents (43.5%) completed the questionnaires at inclusion. At the beginning of the study, 84 (40.8%) participants presented symptoms of burnout, 86 (43.2%) had symptoms of job strain, and 58 (29.4%) of isostrain. Forty-two (20.1%) healthcare workers presented symptoms of sleepiness, and 8 (3.8%) had symptoms of fatigue. We found that symptoms of burnout were more frequent for healthcare workers with a previous psychiatric history (55.3% vs. 39.1%, *p* = 0.02) and were lower among participants who had at least one dependent child (33.1% vs. 48.3%, *p* = 0.013). Symptoms of job strain were higher among administrative staff compared to physicians (55.6% vs. 28.7%, *p* = 0.01) and among healthcare workers with managerial responsibilities compared to those without (45.6% vs. 28.8%, *p* = 0.015). Symptoms of isostrain were higher among administrative staff (42.3%) compared to paramedics (34.1%) and physicians (19.8%, *p* = 0.026).

**Conclusion:** We identified that potential factors associated with the emergence of symptoms of burnout and job strain are suggested, underlining several areas of improvement for the prevention against mental health disorders in the specific population of ED healthcare workers.

**Trial Registration:** ClinicalTrials.gov identifier: NCT04383886.

**Summary**

• During the first outbreak, a large proportion of emergency department's healthcare workers reported symptoms of burnout and job strain but without significant change over time.
• Symptoms of burnout were more frequent in men and in workers with a previous psychiatric history, and administrative staff and healthcare workers having managerial responsibilities were more exposed to symptoms of job strain and isostrain.
• Potential risk factors associated with the emergence of these symptoms are suggested, underlining several areas of improvement for the prevention against mental health disorders in the specific population of ED healthcare workers.

## 1. Introduction

### 1.1. Background

The coronavirus disease 2019 (COVID-19) pandemic was declared on March 11, 2020 [1–3]. In France, the first outbreak started at the beginning of March 2020, which led to a national containment from March 17 to May 11, 2020 [4]. French emergency departments (EDs) were on the frontline, organizing patient triage and managing suspected COVID-19 cases [5]. Before the COVID-19 pandemic and compared to other physicians, emergency healthcare workers were already identified as a vulnerable population regarding burnout, with a reported prevalence of 57% of burnout among French emergency physicians [6]. The same statement was made in the United States by Shanafelt et al., suggesting that the working conditions of the EDs are particularly difficult [7]. The mental health impacts of the COVID-19 pandemic had been studied worldwide demonstrating a strong impact for healthcare workers [8, 9].

### 1.2. Importance

The organizational factors and work environment such as the overwhelming workload, overcrowding, lack of sleep, and accumulated fatigue have indeed been identified by Gualano et al. as risk factors for burnout [10]. Job strain, classically measured using Karasek's job demand-control model, has also been identified as an independent factor associated with burnout [11]. There are also physical consequences. d'Apuzzo et al. demonstrated that a prolonged use of protective face masks for more than 4 h is possibly associated with the presence of discomfort in the preauricular area and headache during the COVID-19 pandemic [12]. Moreover, temporomandibular disorders which are a series of disorders that affect the muscles and joint have been linked by Minervini et al. to psychological factors such as stress, anxiety, and depression [13, 14]. The COVID-19 pandemic added a threat to the mental health of ED workers due to the fear of contamination, the isolation from family and friends, and an increased workload [8, 15, 16]. During the first COVID-19 outbreak, frontline healthcare workers developed more sleep disturbances and worse sleep quality than nonhealthcare professionals [17, 18].

### 1.3. Goals of This Investigation

At the beginning of the pandemic, when the number of cases was rapidly increasing in France, we decided to evaluate the consequences of this pandemic on the mental health of ED healthcare workers. The present study is aimed at providing an assessment of the changes in burnout, job strain, sleepiness, and fatigue levels over time as well as potential risk factors for such symptoms among healthcare workers in French EDs during the first COVID-19 outbreak.

## 2. Methods

### 2.1. Study Design and Setting

This prospective, multicenter, cohort study was conducted in four French EDs and the emergency medical service (*Service d'Aide Médicale Urgente 69*, SAMU) of Lyon, France. The SAMU comprises the mobile emergency and intensive care mobile structures (*Structures Mobiles d'Urgence et de Réanimation*, SMUR) and the emergency call center. The study was approved by the ethics committee Comité de Protection des Personnes Ile-de-France III (CPP, No. 20.04.07.74549) and follows the STROBE statement [19].

### 2.2. Participants and Time Periods Studied

Healthcare workers practicing in EDs or in the SAMU (physicians, paramedics, and administrative staff) during the first outbreak and gave their consent were included from April 20, 2020, to May 29, 2020. The same population was used in both this manuscript and a previous published article [20]. We choose these locations because EDs and the SAMU were representative of frontline healthcare workers during this specific period because all the patients went through the EDs and the SAMU.

### 2.3. Data Collection

#### 2.3.1. Inclusion

Each participant completed a questionnaire at inclusion. The questionnaire addressed demographic characteristics (age, sex, place of residence, marital status, and number of children), history of burnout disorders, use of psychological support, personal organizational characteristics (mode and length of commute, family organization during confinement, and temporary accommodation), and professional characteristics (occupation, type of hospital, years of professional experience, usual work position and reassignments, and managerial responsibility). They also completed specific questionnaires regarding burnout, job strain, sleepiness, and fatigue.

#### 2.3.2. Follow-Up

Each participant completed the questionnaires to assess burnout, job strain, sleepiness, and fatigue at 90 days after inclusion.

#### 2.3.3. Outcomes

We used five outcomes to describe the mental health's impact of the pandemic on burnout, job strain, isostrain, sleepiness, and fatigue. Burnout and sleepiness were chosen because ED healthcare workers represent a population at risk for sleep disorders due to alternating day and night shifts and were already exposed to high level of burnout before the pandemic. Moreover, they were used in other studies, and this allowed comparisons to be made [8]. Job strain, isostrain, and fatigue were less described in the literature, but a relationship between professional stress and burnout had been demonstrated by Velando-Soriano et al. [21].

#### 2.3.4. Burnout

Burnout was evaluated using the validated French version of the Maslach Burnout Inventory (MBI) scale [22], which is composed of 22 items measuring 3 different burnout dimensions: emotional exhaustion (EE; 9 items), depersonalization (DP; 5 items), and personal achievement (PA; 8 items) [23]. The total score of each dimension was classified as low, moderate, or high, according to the cutoffs previously published [23]. Respondents with high levels of EE (> 26) and/or DP (> 12) and/or or low levels of PA (< 29) were considered with burnout.

### 2.4. Job Strain and Isostrain

Job strain was measured using the job control-demand model from Karasek, which is widely used to evaluate psychosocial factors at work [24, 25]. The French version of the Karasek questionnaire was validated by Brisson et al. in 1998 [26]. The questionnaire includes 26 items to assess 3 dimensions: decision latitude (9 items), psychological job demand (9 items), and social support (8 items). For each item, the subject is asked to respond using a 4-level Likert-type scale. A score below 20 reflects a low psychological job demand. A score below 71 reflects low decision latitude. A score below 24 reflects low social support. Job strain is the combination of low decision latitude and high psychological job demand. In practice, if the psychological job demand score is higher than 20 and the decision latitude score is less than 71, the employee is in the tense quadrant and therefore considered to suffer from job strain which constitutes a health risk situation [27, 28]. Isostrain is the combination of job strain and low social support (score below 24) [29].

### 2.5. Sleepiness

Sleepiness was evaluated using the Epworth Sleepiness Scale (ESS) which is a self-administered questionnaire composed of eight questions by Kaminska et al. [30]. Respondents were asked to rate, on a 4-point scale (0–3), their usual chances of dozing off or falling asleep while engaged in eight different activities. Most people engage in those activities at least occasionally, although not necessarily every day. The ESS score (the sum of the eight-item scores, 0–3) can range from 0 to 24. The higher the ESS score, the higher the subject's average sleep propensity in daily life, or their daytime sleepiness. ESS scores of 11–24 represent levels of excessive daytime sleepiness [31]. The French version of this tool has also been validated [30].

### 2.6. Fatigue

Fatigue was assessed using the Pichot Fatigue Scale (PFS) [32], which is a self-administered questionnaire composed of eight questions. Fatigue is a feeling of physical or mental weakness that normally occurs after a sustained effort and which requires rest. Pathological fatigue is considered when a subject feels handicapped compared to his or her usual level of fitness to perform daily activities. Respondents were asked to rate their fatigue according to eight questions on a 5-point scale, from 0 (not at all) to 4 (extremely). The PFS score can range from 0 to 32; a score > 22 is in favor of excessive fatigue [32].

### 2.7. Statistical Analysis

Continuous data were described by means (± standard deviation) and categorical ones by frequencies and percentages. The results from the different scales were binarized according to their respective cutoffs to analyze a possible alteration in the scale between inclusion and Day 90. These were then modelled by a logistic mixed effect model including a period effect and the fortnight week of inclusion since the beginning of the study and a random intercept by healthcare worker. The change in the scales over time was quantified by odds ratios with their associated 95% confidence interval (95% CI). The analyses of factors associated with the different scales were performed using the same model, by adding the factors one at a time into the model. The model was compared with and without the factor using a likelihood-ratio test, to assess the overall effect of the factor. In this mixed model taking into account multiple measurements, results from the scales at inclusion and Day 90 were pooled together in order to increase sample size. For some factors however, due to the low number of respondents presenting symptoms on certain scales, simple chi-squared or Fisher's exact tests were performed. No correction for multiple testing was performed. *p* values less than 0.05 were considered significant. The analyses were performed using the R software.

## 3. Results

In the study, among the 485 healthcare workers asked to participate, 211 (43.5%) respondents completed the survey at inclusion. Out of 210 respondents, 144 (68.6%) worked in EDs and 116 (55.2%) were paramedics. During the crisis, 16 (7.6%) participants had been reassigned to frontline departments. A total of 41 (19.7%) healthcare workers had managerial responsibilities (Table 1). Nineteen (9%) reported having a previous burnout, and among them, only 2 (10.5%) participants reported using a treatment.

### 3.1. Change Over Time in Burnout, Job Strain, Isostrain, Sleepiness, and Fatigue

At inclusion, 84 (40.8%, 95% CI: [34.0%, 47.8%]) participants presented symptoms of burnout. Symptoms of job strain and isostrain were found for 86 (43.2%, 95% CI: [36.2%, 50.4%]) and 58 (29.4%, 95% CI: [23.2%, 36.3%]) healthcare workers, respectively. Forty-two (20.1%, 95% CI: [14.9%, 26.2%]) healthcare workers had symptoms of sleepiness, and only 8 (3.8%, 95% CI: [1.7%, 7.4%]) had symptoms of fatigue. Although there was a tendency towards increased sleepiness overtime (OR 2.11, 95% CI [0.67, 6.50]), there was no significant change in the frequency of the different symptoms between inclusion and Day 90 (Table 2).

### 3.2. Factors Associated With Burnout, Job Strain, Isostrain, and Sleepiness

Symptoms of burnout were significantly higher among men compared to women (48.0% vs. 36.6%, *p* = 0.028, respectively) and more frequent for healthcare workers with a previous psychiatric history (55.3% vs. 39.1%, *p* = 0.02). Conversely, symptoms of burnout were lower among participants having at least one dependent child (33.1%) compared to those without dependent children (48.3%, *p* = 0.013).

Symptoms of job strain were statistically different depending on the occupation (*p* = 0.001), with statistical difference between paramedics (50.6%) and physicians (28.7%, *p* = 0.012). Job strain was more present among healthcare workers with managerial responsibilities (45.6%) compared to those without (28.8%, *p* = 0.015).

Symptoms of isostrain were also statistically different depending on the occupation (*p* = 0.026), with statistical difference between paramedics (34.1%) and physicians (19.8%, *p* = 0.014). Isostrain was more present among healthcare workers with a professional experience of 6–10 years (42.0%) compared to those with a professional experience of 0–2 years (22.1%, *p* = 0.036). No factor was statistically associated with sleepiness (Table 3).

## 4. Discussion

During the first outbreak, a large proportion of ED healthcare workers reported symptoms of burnout and job strain but without significant change over time. To a lesser extent, symptoms of isostrain and sleepiness were also present in one-third of the respondents. Symptoms of burnout were more frequent in men and in workers with a previous psychiatric history but less frequent in respondents who had a dependent child. The administrative staff and healthcare workers having managerial responsibilities were more exposed to symptoms of job strain and isostrain.

### 4.1. Burnout Symptoms

Although the present results confirm the finding that emergency healthcare workers face symptoms of burnout, the frequency of burnout was lower herein compared to the about 60% previously reported [6, 7, 33]. During the COVID-19 pandemic, 74.7% of burnout was reported among US emergency medicine physicians [15] and a review reported a prevalence of overall burnout ranging from 49.3% to 58% among healthcare professionals of intensive care units and EDs [10]. Interestingly, it has been reported that the pandemic was not necessarily associated with increased symptoms of burnout among general healthcare workers [8] and that healthcare workers who directly addressed the virus had decreased chances of burnout occurrence [34]. In the present study, the proportion of respondents displaying symptoms of burnout did not increase over time, suggesting that the healthcare workers were able to cope during this first outbreak of the COVID-19 pandemic. Among Canadian emergency physicians, burnout levels also remained stable during the first 10 weeks of the pandemic but increase over time [35]. The same authors, however, found an association between being tested positive for COVID-19 and symptoms of EE and DP, a finding which was not confirmed herein [36]. Although paramedics have been identified by Chor et al. as a vulnerable population in terms of burnout occurrence, the current study could not confirm this finding [37]. The high prevalence of burnout symptoms among ED staff reported herein further underlines the need to prioritize the mental health of ED healthcare workers, particularly since it has been shown that these symptoms can start as early as the residency training [38]. A major difficulty, however, is that the trigger factors for developing burnout symptoms in emergency medicine are plural and diverse [39].

### 4.2. Job Strain and Isostrain

The prevalence of job strain found herein was higher than the 27.1% reported in French EDs before the COVID-19 pandemic [40]. According to the present results, job strain was more frequent than isostrain in French ED healthcare workers, and both were more frequent among administrative staff compared to physicians and paramedics. One hypothesis is that the administrative staff, who do not provide patient care, are less integrated with healthcare workers in EDs and have thus less support from the healthcare team. A predictive effect of psychosocial factors at work on the development of anxiety-depressive symptoms in employees has been demonstrated by Niedhammer et al. [29]. This provide an overview on the impact on the mental health of ED healthcare workers during the critical period of the pandemic.

### 4.3. Sleepiness

Although ED healthcare workers represent a population at risk for sleep disorders due to alternating day and night shifts, sleepiness in this particular population has rarely been studied. Herein, a relatively important proportion (about a fifth) of healthcare workers presented symptoms of sleepiness. It was shown that during the COVID-19 pandemic, frontline healthcare workers experienced sleep disturbances in a more frequent manner than second-line healthcare workers [17, 18]. Work shift has been associated with a greater risk of experiencing these symptoms [31]. Although no factor associated with sleepiness was identified herein, symptoms of sleepiness, unlike those of burnout, job strain, and isostrain, tended to increase over time, possibly due to a cumulative effect for these symptoms.

### 4.4. Fatigue

Few healthcare workers presented symptoms of fatigue in our study, while nearly a fourth had excessive sleepiness at Day 90, confirming that sleepiness and fatigue are two interrelated but distinct phenomena [41]. Fatigue is often considered as a feeling of physical or psychological exhaustion induced by an intense and sustained effort [41]. Sleepiness distinguishes itself from fatigue by a presumed impairment of the normal arousal mechanism [41]. Several parameters may have prevented caregivers from experiencing fatigue during the first COVID-19 outbreak. The multiple following outbreaks, however, might have impacted this state of fatigue. Oțelea et al. found that age, imbalance between effort and reward, overcommitment, and management of the risk of infection in the workplace were associated with the fatigue score in healthcare workers after the first three waves of the COVID-19 pandemic [42]. Management of fatigue in EDs is crucial, particularly since multiple shifts that include night shifts increase the risk of developing severe chronic fatigue syndromes and burnout [43].

### 4.5. Implications and Strategies to Improve Healthcare Workers' Mental Health

These results provide opportunities to think how healthcare institutions and policymakers can support the mental health of ED workers during the ongoing pandemic and in similar situations. Indeed, Chemali et al. highlighted that the effects of the pandemic on healthcare workers are closely related to individual, interpersonal, and also institutional aspects [44]. Alfonsi et al. have demonstrated the urgent need for preventive programs among healthcare operators to increase their coping skills and prevent the long-term consequences of chronic stress, especially for high-risk professionals [9]. Lee et al. also underlined the need of targeted intervention depending towards the differences between at risk groups to decrease the mental health's long-term consequences [8]. For example, specific attention should be devoted to programs to improve sleep quality especially for population at risk for sleep disorders due to alternating day and night shifts such as ED healthcare workers. Improving the organization of working times and increasing the social support for ED healthcare workers can also reduce the impact on job strain and isostrain. Finally, our results underline the need to prioritize interventions to reduce burnout and improve well-being as it had been suggested by Chor et al. [37] and the necessity of regular assessment of the mental health of ED healthcare workers over time in order to adapt the strategies.

### 4.6. Limitation

The present study has several limitations. First, the study was only conducted during the first outbreak. Since then, the several outbreaks of COVID-19 cases that occurred in France could have led to the exhaustion of healthcare workers. Second, we had a low response rate (43.5%), and moreover, only two-thirds of the participants completed the questionnaires at the 90-day follow-up, which may introduce selection bias. The professionals who did not complete the study and also those who did not give their consent for participate might have been the most at risk for burnout, leading to a possible underestimation of the levels of burnout, job strain, isostrain, sleepiness, and fatigue. It also impacts the generalizability of the findings of the study. The study's results on job stress, burnout, and sleepiness may also be influenced by different hospitals, countries, and policies, which could limit generalizability. Finally, since it was not possible to analyze associations between factors and the different scales in fully adjusted models, conclusions should thus be confirmed in other studies. Moreover, due to the limited sample size, it was not possible to perform multivariable analyses by including all the factors simultaneously into the same model; one model was built for each factor, and adjustment was performed only on period and fortnight of inclusion. Nevertheless, future studies should take these limitations into consideration. Including different countries and different hospitals but also more participants could help to generalize the result and perform multivariable analysis.

## 5. Conclusion

During the first COVID-19 outbreak, a high proportion of ED healthcare workers presented mental health consequences. Potential risk factors associated with the emergence of these symptoms are suggested, underlining several areas of improvement for the prevention against mental health disorders in the specific population of ED healthcare workers. Further studies are needed to assess the long-term mental health consequence of the pandemic and to evaluate strategies in order to decrease burnout and improve well-being as well as the quality of sleep.

## Figures and Tables

**Table 1:** Demographic and occupational characteristics of respondents.

**Characteristics**	**Total (** **N** ** = 211)**
Occupation (*n* = 210)	
Physicians	76 (36.2)
Paramedics	116 (55.2)
Administrative staff	18 (8.6)
Age, years (*n* = 210)	37.4 ± 9.6
Women	150 (71.1)
Marital status (*n* = 210)	
Married or in a relationship	159 (75.7)
Single	51 (24.3)
Dependent child (*n* = 210)	110 (52.4)
Years of professional experience (*n* = 209)	11.1 ± 9.0
Usual work position (*n* = 210)	
Emergency department	144 (68.6)
Emergency medical service	32 (15.2)
Both emergency department and emergency medical service	18 (8.6)
Other unit (reassignment)	16 (7.6)
Managerial responsibility (*n* = 208)	41 (19.7)

*Note:* Data are expressed as *N* (%), or mean ± SD.

Abbreviation: SD, standard deviation.

**Table 2:** Change over time in burnout, job strain, isostrain, sleepiness, and fatigue among emergency healthcare workers.

	**Inclusion** **N** ** = 211**	**Day 90** **N** ** = 128**	**OR [95% CI]**	**p** ** value**
Burnout (yes)	84/206 (40.8%)	47/121 (38.8%)	0.90 [0.50; 1.61]	0.719
Job strain (yes)	86/199 (43.2%)	49/119 (41.2%)	0.96 [0.54; 1.69]	0.878
Isostrain (yes)	58/197 (29.4%)	34/118 (28.8%)	1.03 [0.58; 1.83]	0.918
Sleepiness (yes)	42/209 (20.1%)	29/121 (24.0%)	2.11 [0.67; 6.50]	0.193
Fatigue (yes)	8/210 (3.8%)	5/121 (4.1%)	^a^	0.683

*Note:* Odds ratios (OR) of event between inclusion and Day 90 (analysis adjusted on the fortnight of inclusion) are presented.

Abbreviation: CI: confidence interval.

^a^OR not available due to small number of healthcare workers with fatigue.

**Table 3:** Factors associated with burnout, job strain, isostrain, and sleepiness among emergency healthcare workers.

**Variable**	**Burnout**	**p**	**Job strain**	**p**	**Isostrain**	**p**	**Sleepiness**	**p**
Occupation		0.093		0.001		0.026		0.920
Administrative staff	7 (25.9%)		15 (55.6%)		11 (42.3%)		3 (11.1%)	
Paramedics	67 (38.5%)		85 (50.6%)		57 (34.1%)		39 (22.2%)	
Physicians	57 (45.6%)		35 (28.7%)		24 (19.8%)		29 (23.0%)	
Sex		0.028		0.260		0.313		0.612
Women	83 (36.6%)		99 (44.8%)		69 (31.4%)		54 (23.6%)	
Men	48 (48.0%)		36 (37.1%)		23 (24.2%)		17 (16.8%)	
Marital status		0.314		0.511		0.753		0.976
Married/relationship	94 (38.4%)		99 (41.2%)		24 (31.2%)		53 (21.4%)	
Single	37 (45.7%)		36 (46.8%)		68 (28.7%)		18 (22.2%)	
Dependent child		0.013		0.488		0.291		0.671
Yes	58 (33.1%)		72 (41.1%)		47 (27.3%)		36 (20.0%)	
No	73 (48.3%)		63 (44.4%)		45 (31.7%)		35 (23.5%)	
Temporary accommodation		0.917		0.127		0.223		0.129
Yes	4 (36.4%)		7 (63.6%)		5 (45.5%)		0 (0.0%)	
No	125 (39.9%)		126 (41.4%)		87 (28.9%)		69 (21.8%)	
Usual work position		0.273		0.255		0.784		0.109
Emergency medical service (SAMU)	14 (28.0%)		27 (55.1%)		14 (29.2%)		10 (19.6%)	
Emergency department (ED) (*n* = 222)	91 (41.0%)		92 (42.6%)		64 (29.8%)		43 (19.2%)	
Both ED and SAMU	16 (55.2%)		10 (35.7%)		9 (33.3%)		11 (37.9%)	
Reassignment	10 (40.0%)		6 (25.0%)		5 (20.8%)		7 (28.0%)	
Professional experience, years		0.524		0.288		0.036		0.111
0–2	38 (38.4%)		38 (39.6%)		21 (22.1%)		18 (18.4%)	
3–5	25 (51.0%)		21 (45.7%)		14 (30.4%)		16 (33.3%)	
6–10	30 (41.7%)		35 (50.0%)		29 (42.0%)		12 (16.2%)	
≥ 10	37 (36.3%)		40 (39.6%)		27 (27.0%)		25 (23.8%)	
Previous psychiatric history		0.002		0.447		0.450		0.999
Yes	42 (55.3%)		32 (45.1%)		22 (31.4%)		16 (31.3%)	
No	117 (39.1%)		103 (41.9%)		70 (28.7%)		55 (21.7%)	
COVID-19 infection		0.274		0.994		0.563		1.000
Yes	14 (50.0%)		12 (44.4%)		7 (25.9%)		5 (17.9%)	
No	117 (39.1%)		123 (42.2%)		85 (29.7%)		66 (21.9)	
Work disability related to infection or that of a loved one		0.445		0.364		0.293		0.878
Yes	22 (45.8%)		16 (35.6%)		10 (22.7%)		10 (208%)	
No	109 (39.5%)		119 (44.1%)		82 (30.6%)		59 (21.1%)	
Managerial responsibility		0.795		0.015		0.080		0.899
Yes	28 (40.6%)		19 (28.8%)		13 (20.0%)		17 (25.0%)	
No	102 (40.2%)		113 (45.6%)		77 (31.3%)		54 (20.9%)	

*Note:* The number of respondents and results from the different scales at inclusion and Day 90 were pooled together in order to increase sample size; *p* values were obtained from mixed models that took into account multiple measurements and correspond to overall test.

## Data Availability

The datasets used and/or analyzed during the current study are available from the corresponding author on reasonable request.

## Nomenclature

COVID-19: the coronavirus disease 2019
EDs: emergency departments
ESS: Epworth Sleepiness Scale
MBI: Maslach Burnout Inventory
PFS: Pichot Fatigue Scale
SAMU: emergency medical service (Service d'Aide Médicale Urgente)
